# Pediatric Osteoarticular Tuberculosis as a Diagnostic Dilemma and a Review of Literature

**DOI:** 10.7759/cureus.23053

**Published:** 2022-03-11

**Authors:** Sumit Gupta, Asmita Parihar, Savitri Singh, Ankur Agarwal, Sheetal Agarwal

**Affiliations:** 1 Orthopedics, Lady Hardinge Medical College, New Delhi, IND; 2 Pathology, Super Religare Laboratories, Ghaziabad, IND; 3 Pathology, Super Speciality Pediatric Hospital & Post Graduate Teaching Institute, Noida, IND; 4 Pediatric Orthopedics, Super Speciality Pediatric Hospital & Post Graduate Teaching Institute, Noida, IND; 5 Pediatrics, Atal Bihari Vajpayee Institute of Medical Sciences and Dr. Ram Manohar Lohia Hospital, New Delhi, IND

**Keywords:** mimicking, atypical presentation, pediatric, bones and joints, dilemma, diagnostic challenge, osteoarticular, extrapulmonary, tb, tuberculosis

## Abstract

Introduction

Despite tuberculosis being rampant in the Indian subcontinent, most cases of osteoarticular (OA) tuberculosis (TB) are missed until significant bony destruction has occurred. Initial presentation of extra-pulmonary TB mimics many other disease entities while many diseases mimic TB. This may lead to an incorrect diagnosis and sometimes creates a dilemma in reaching the correct diagnosis. The aim of this study was to evaluate a series of pediatric cases of osteoarticular TB, which posed a diagnostic challenge to us.

Material and methods

Retrospective analysis of case records of pediatric OA-TB patients who had presented to two tertiary level centers of urban India between February 2016 and December 2020 was done. There were a total of 69 patients.

Observations

There were 37 males and 32 females. The age range was from two to 17 years. Forty-four patients showed evidence of disease within the spine (dorsal region followed by lumbar, followed by the cervical spine), 16 showed disease of the extremities, six had disease of the girdle bones, and three showed disease of the short bones of hands or foot. In our series, patients presented to us between 15 days to six months from the onset of symptoms. From our series, six cases with atypical clinical pictures have been selected for presentation purposes. In all six cases, the initial presentation was not that of OA-TB. However, with a high degree of suspicion, differential diagnosis of TB was kept in mind, and the diagnosis was confirmed microbiologically.

Conclusion

A high degree of suspicion is required to avoid missing the diagnosis of osteoarticular TB. Non-invasive advanced radiological investigations such as MRI and microbiological analysis of biopsy specimens aid in arriving at the correct diagnosis.

## Introduction

Tuberculosis (TB) is rampant in the Indian subcontinent, with an incidence of 193 new cases each year per 100,000 population. Despite this, most cases of osteoarticular tuberculosis are not definitely diagnosed until significant bony destruction has occurred because of varied presentation - approximately 20% of all mycobacterial infections in children present as extra-pulmonary TB (EPTB). In the pediatric age group, tuberculosis of bones or joints occurs in 5% in cases of EPTB [1]. Initial presentation of EPTB mimics many other disease entities while many diseases mimic TB, such that TB has been termed ‘a great imitator’ [2,3]. Infections such as fungi, brucellosis, sarcoidosis, and mycobacterium leprae have many common clinical and radiological features with osteoarticular (OA) TB. Diabetes, immune deficiency diseases, immune-suppressant drugs, chronic liver, and renal diseases further complicate the picture [4]. This may lead to an incorrect diagnosis and could create a dilemma in arriving at the correct diagnosis. Delayed diagnosis is more common in the pediatric age group because of lack of awareness of the condition, nonspecific clinical symptoms, and non-characteristic imaging findings [4]. Constitutional symptoms may be absent in up to 72% of cases [5].

The aim of this study was to evaluate a series of pediatric OA-TB cases of which many patients had ab atypical presentation or presented to us as a diagnostic dilemma.

## Materials and methods

The case records of pediatric OA-TB patients who had presented to two tertiary level centers of urban India between February 2016 and December 2020 were included in the study. There were a total of 69 patients, of which 37 were males and 32 were females. The age range was from two to 17 years. The site distribution of the cases is presented in Table 1.

**Table 1:** Distribution of cases by affected site

Site	Males (n=37)	Females (n=32)	Total (n=69)
Cervical spine	4	3	7
Dorsal spine	9	16	25
Lumbosacral spine	8	3	11
Spine (multifocal/ skip lesions)	0	1	1
Hip region	6	2	8
Knee region	2	1	3
Elbow region	1	1	2
Ulna	2	0	2
Wrist region	1	0	1
Shoulder girdle (clavicle/ scapula)	2	3	5
Pelvic girdle (ilium)	1	0	1
Short bones of hands	0	1	1
Short bones of foot	1	1	2

Forty-four patients had affection of the spine (dorsal region followed by lumbar, followed by the cervical spine), 16 had affection of the extremities, six had affection of the girdle bones, while three had affection of the short bones of hands or foot. In our series, patients presented to us between 15 days to six months from the onset of symptoms. 

Out of the 69 patients, we selected six patients in whom tuberculosis was mimicking with other diseases, and presentation was uncommon for the detailed presentation. The criterion for uncommon presentation was either the absence of typical constitutional symptoms associated with tuberculosis, a clinical picture mimicking other disease entities, a radiological picture of some other osteoarticular disease, or a rare site of occurrence. In each patient, an attempt was made to obtain material by open biopsy for microbiological assessment. Gram staining, Ziehl Neelsen staining, Auramine staining, Bacterial culture sensitivity, automated TB culture, and Genexpert-MTB were carried out on the material that was obtained from a biopsy.

Anti-tubercular drug therapy (ATT) was given through directly observed therapy short-course (DOTS) under the Revised National Tuberculosis Control Programme (RNTCP) for 12 months in each case. Monitoring of patient weight, liver function tests, renal function tests, complete blood count, ESR and plain radiography of the affected part was done at regular intervals. Patients were also clinically assessed regularly, including an ophthalmologic examination. Any side-effect of anti-tubercular chemotherapy was also noted. As per the latest RNTCP guidelines, all patients were put on regular follow-up up to one year after completion of ATT.

## Results

Case one - clinical dilemma

**Figure 1:**
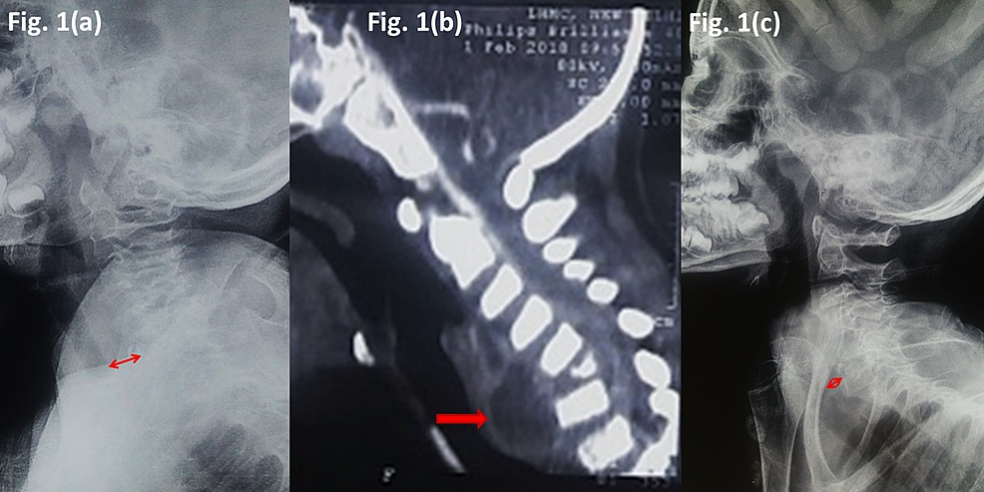
Cervical spine TB with cerebral palsy (a) Plain radiograph at presentation (b) CT scan (c) After 12 months of ATT TB - tuberculosis, ATT - anti-tubercular drug therapy

A seven-year-old boy presented with irritability and a low-grade fever for 15 days. He was a known case of cerebral palsy, documented as Gross Motor Function Classification System (GMFCS) level III. He was non-verbal with speech problems. He was on anti-epileptic medication. On examination, the child was not able to sit and conformed to GMFCS Level IV. Tone was increased in all four limbs, and deep tendon reflexes were exaggerated. Plantar reflex was upgoing. However, the child was able to communicate with the caregiver with eye contact. A provisional clinical diagnosis of meningitis was made. Cerebrospinal fluid (CSF) examination was normal. Biochemical parameters were equivocal. A plain radiograph of the neck raised suspicion of the C6 vertebra. A CT scan was done, which confirmed the destruction of the C6 vertebra along with a large pre-vertebral abscess (Figure 1). 

Anterior decompression of the cervical spine (Video 1) with a fusion of the C5-C7 vertebrae using fibular graft was done. 

**Video 1:** Anterior decompression of the cervical spine

Material obtained from decompression confirmed tuberculosis. Cervical traction was applied for four weeks, and the GMFCS level of the child improved back from IV to III. Anti-tuberculous therapy (ATT) was continued for 12 months. The patient was discharged on a soft cervical collar and was able to sit upright (Video 2). 

**Video 2:** Clinical follow-up

The patient was followed up for a period of 36 months from the start of ATT. He was able to stand with support but was not able to walk at the last follow-up.

Case two - radiological dilemma

**Figure 2:**
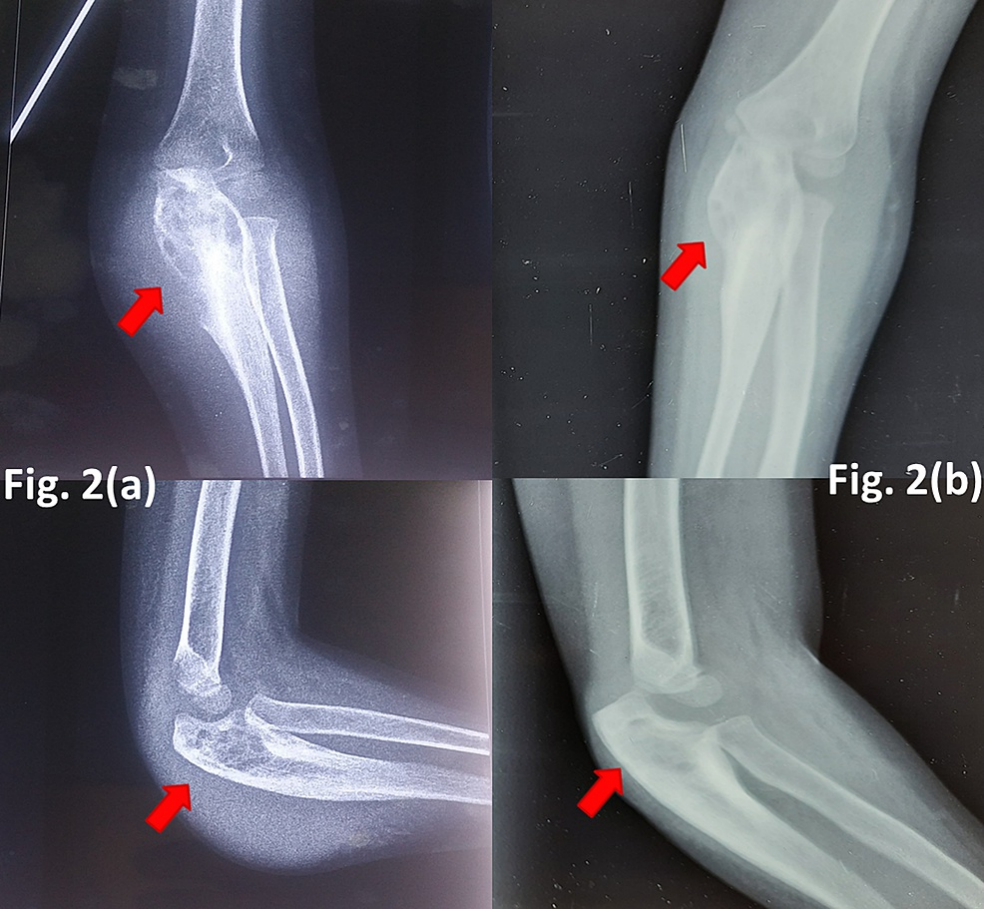
TB of the olecranon process of ulna (a) Plain radiograph at presentation (b) At 15 months follow-up TB - tuberculosis

A four-year-old girl presented with a swelling in the left elbow region for the past two months. A plain radiograph showed an expansile lytic lesion in proximal ulna with irregular bony destruction. A provisional diagnosis of fibrous dysplasia was made based upon radiographic findings. Open biopsy was taken, which confirmed the diagnosis of tuberculosis histologically. ATT was given for 12 months. The lesion showed signs of resolution at the last follow-up, which was 24 months post start of ATT (Figure 2).

Case three - radiological dilemma

**Figure 3:**
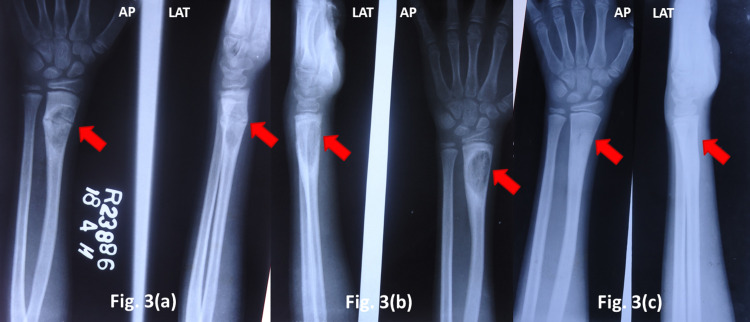
TB of distal end radius (a) Plain radiograph at presentation (b) After eight months on ATT (c) At 18 months follow-up TB - tuberculosis, ATT - anti-tubercular drug therapy

An eight-year-old boy presented with pain and swelling of the right wrist for two months. Tenderness was localized over thedistal radius. Plain radiography revealed a well-circumscribed lytic lesion in the distal radius with a pathological fracture. Radiologically, the lesion appeared similar to a simple bone cyst or an aneurysmal bone cyst. An open biopsy revealed histological features suggestive of a tuberculous infection. The fracture was splinted in plaster and ATT was prescribed for 12 months. At 18 months post the start of ATT, there were signs of healing of the lesion (Figure 3). 

Case four - radiological dilemma

**Figure 4:**
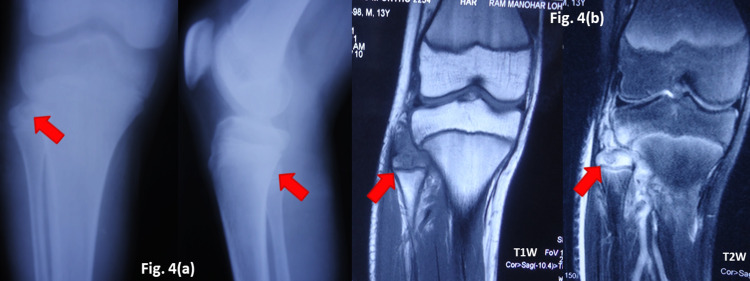
TB of the proximal end of fibula (a) Plain radiograph at presentation (b) MRI (T1-weighted and T2-weighted images) TB - tuberculosis

A 12-year-old boy presented with a slowly growing swelling at the proximal fibula in the right leg for three months. Plain radiographs revealed a lytic lesion in the proximal fibular epiphysis mimicking a chondroblastoma. Magnetic resonance imaging (MRI) showed intra-osseous abscess with soft tissue extension, raising suspicion for OA-TB. Hematological parameters also pointed towards an infective lesion. After a biopsy, which confirmed the diagnosis, ATT was started, and the lesion showed signs of resolution by eight months. ATT was continued for the full 12-month duration. The last follow-up of the patient was at 20 months from the start of ATT (Figure 4).

Case five - rare site

**Figure 5:**
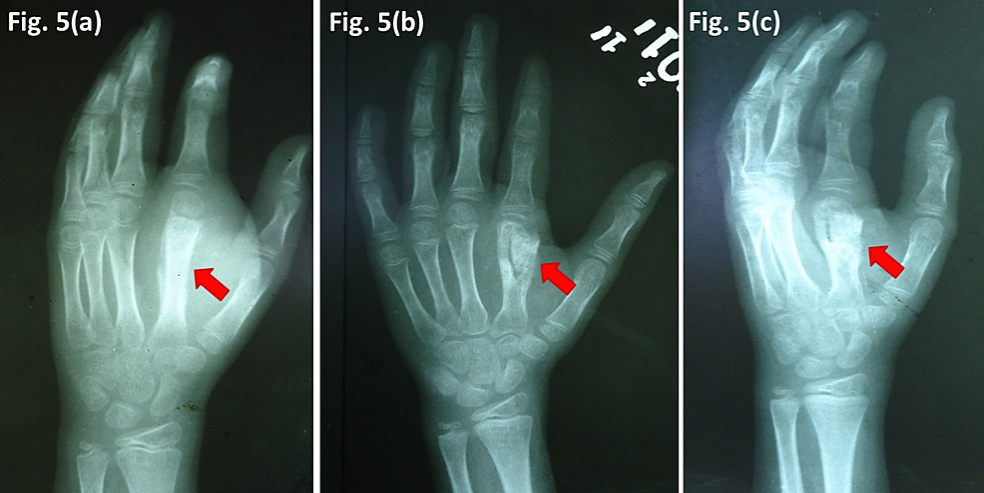
TB of metacarpal and phalanx (a) Plain radiograph taken elsewhere three months before presentation (b) At two months post ATT (c) After 12 months ATT TB - tuberculosis, ATT - anti-tubercular drug therapy

A 9-year-old girl presented with a tense 4 cm x 3 cm swelling and a non-healing ulcer over the second metacarpal of the right hand for the past four months. She was being treated in the periphery with antibiotics and non-steroidal anti-inflammatory drugs. Plain radiographs showed a lytic lesion of bone with ballooning and thinning of the cortex, which mimicked chronic osteomyelitis. Careful observation also showed a sequestrum. The possibility of spina ventosa, was kept in mind. MRI findings were consistent with an infective lesion. Subsequently, an open biopsy was carried out, which confirmed the diagnosis of OA-TB microbiologically. ATT was given for 12 months, to which the patient responded well (Figure 5).

Case six - rare site

**Figure 6:**
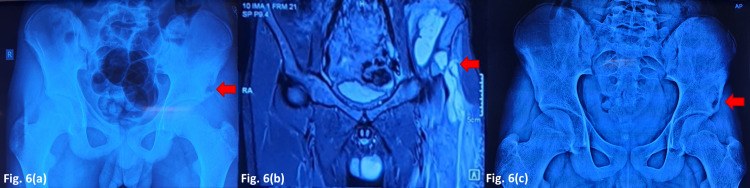
TB of the iliac bone (a) Plain radiograph at presentation (b) MRI (T2-weighted image) (c) After 12 months ATT TB - tuberculosis, ATT - anti-tubercular drug therapy

A 16-year-old otherwise healthy boy presented with pain and swelling in the left inguinal region present for the past six months, which was being treated as osteomyelitis with antibiotics. There was no discharging sinus. Hematological parameters were equivocal. Plain radiographs showed a lytic lesion near the left anterior inferior iliac spine. MRI revealed bony destruction with collection suggestive of infective pathology. Incisional biopsy was carried out which on gross examination revealed caseous material admixed in watery purulent material. Cartridge-based nucleic acid amplification test (CBNAAT) was positive for mycobacterium TB. Ziehl-Neelsen (ZN) staining was negative, but auramine staining showed few tubercle bacilli. ATT was given for 12 months, with radiological signs of resolution at the last follow-up (Figure 6).

## Discussion

These six cases of osteoarticular tuberculosis represent a spectrum of osteoarticular tuberculosis, which has the potential to create a diagnostic dilemma. The duration of symptoms in these patients varied between 15 days to six months as the patients were being treated in a peripheral unit for an alternative diagnosis based on clinico-radiological grounds. TB can have a varied presentation, and therefore it is essential to keep the tubercular infection in differential diagnosis while working up for any infective bony pathology. Not only this, OA-TB should be kept as a differential in any pathology where there is lysis of bone in a plain radiograph.

The most common reason why a diagnosis of osteoarticular tuberculosis is delayed is that the usual presenting complaints are vague and are seen in many diseases. Initially, there is only pain with or without associated swelling. As a result, a patient with osteoarticular tuberculosis can reach many advanced stages such as pathological fractures, non-unions, arthritis, ankylosis, instability, limb deformity, pathological dislocations, osteomyelitis, neurological deficit, and avascular necrosis. These can present as a first symptom or sign when the patient reaches an orthopedic surgeon or until the final diagnosis is arrived at. Other atypical presentations include acute form, cystic form, multifocal infliction in the same patient, and epiphyseal and metaphyseal osteomyelitis [4]. Less commonly, the atypical presentation can be in the form of affection of unusual anatomical locations like the sternoclavicular joint, acromioclavicular joint, clavicle, scapula, symphysis pubis, skull, facial bones, sternum, ribs, etc. Since the location of affection is not usual, a high degree of suspicion is required to proceed for a detailed workup to arrive at the diagnosis [6,7].

In our series, cervical spine tuberculosis (case one) upper motor signs were masked by a spasm and contractures of cerebral palsy, so the diagnosis of tuberculosis was delayed. In three patients (cases two, three, and four), radiological picture mimicked a benign tumor. Fracture in a tubercular lesion is quite rare. Most of the cases of fractures in tubercular lesions are reported in the spine or lower limbs. These fractures heal spontaneously with ATT [8]. In two patients (cases five and six), the site of presentation was rare. The long bones, especially of the lower limbs in children, are more affected than the pelvis and axial skeleton in contrast to adult osteoarticular tuberculosis. Pediatric osteoarticular TB can be located in the metaphyseal region or in the epiphysis or can extend from the metaphysis to involve growth plate due to transphyseal spreading. Multiple sites of bony destruction within the same bone and skip lesions are more common in children, while single bone involvement is more usual in adults. Four types of plain radiographic features of TB osteomyelitis can be seen. These include lucency, focal erosions, infiltrative lesions, and ballooning of bone cortices. As compared to pyogenic osteomyelitis, TB osteomyelitis lacks sclerosis and has fewer occurrences of sequestrum and periosteal reaction. Children also tend to have less frequent sclerotic bony destruction than adults. OA-TB can mimic Ewing sarcoma, fungal and subacute pyogenic osteomyelitis, cartilaginous tumors, or Langerhans cell histiocytosis. TB dactylitis, or spina ventosa, is most commonly found in infants and young children [9,10]. 

Several authors have presented the diagnostic challenges of osteoarticular TB (Table 2) [11-23]. Our study summarizes and categorizes the reasons behind the dilemma.

**Table 2:** Review of literature on diagnostic dilemmas in OA-TB FNAC - fine needle aspiration cytology, TB - tuberculosis, OM - osteomyelitis, GCT - giant cell tumor, PVNS - pigmented villonodular synovitis, AVN - avascular necrosis, PAN - poly-arteritis nodosa, OA - osteoarthritis, RA - rheumatoid arthritis, PCR - polymerase chain reaction, OA-TB - osteoarticular tuberculosis

S.No.	Researcher	Number of cases	Site	Confusing factor	Initial diagnosis/ differential diagnosis	Aids to diagnosis
1	Agarwal et al. 2021 [4]	1	Phalanx	Discharging sinus	Chronic OM	Biopsy smear
2	Elghoul N et al. 2020 [12]	2	Bilateral shoulders; ilium bone	Multifocal symptoms; rare site with congenital malformations	Multiple myeloma	Biopsy smear, PCR
3	Wagh et al. 2020 [13]	25	Long bones of extremities	Radiological picture	Tumors	Biopsy smear
4	Reddy et al. 2018 [14]	1	Tendon synovial sheath of index finger	FNAC picture	GCT tendon synovial sheath	Biopsy smear
5	Vijay et al. 2015 [15]	2	Calcaneo-cuboid joint	Rare site	-	Biopsy smear
6	Birjandinejad et al. 2012 [16]	1	Talonavicular joint	Rare site, ulceration	Leishmaniasis	Biopsy smear
7	Sheikh et al. 2012 [17]	1	Condyle of mandible	Atypical clinical and radiological picture	Chronic suppurative osteomyelitis and fungal osteomyelitis	Biopsy smear
8	Choi et al. 2008 [18]	15	Forefoot(2), midfoot(3), and ankle(10)	Atypical clinical and radiological picture	Pyogenic osteomyelitis, PVNS, amyloidosis, AVN	Biopsy smear, culture, PCR
9	Winsnes et al. 2005 [19]	5	Spinal TB (lumbosacral)	Rare/ forgotten disease in Norway	Sciatica	Biopsy smear
10	Sih et al. 2004 [20]	1	C1-C2 vertebra	Atypical clinical presentation	Meningioma, metastasis	Biopsy smear
11	Paull et al. 1999 [21]	4	Disseminated multisystem disease	Atypical clinical presentation	Metastasis, lymphoma, PAN, cholecystitis, depression	TB culture
12	Nussbaum et al. 1995 [22]	29	Spinal TB	Varied clinical picture	Pyogenic osteomyelitis, metastasis	Biopsy smear, culture
13	Ellis et al. 1993 [23]	15	Peripheral joints	Varied clinical picture	OA, RA, pyogenic arthritis, reactive arthritis	Biopsy smear, synovial fluid smear, culture

The dilemma can be in the form of an unclear clinical picture, radiological misinterpretation with other disease entities, or caused due to affection of a rare site of occurrence. With a high degree of suspicion on clinico-radiological grounds, advanced non-invasive radiological investigations should never be deferred. Magnetic resonance imaging (MRI) throws light on many findings which are not obvious on plain radiography. Marrow edema, rim enhancement on T2-weighted images, signal intensity changes, collection within or around the bone destruction, occult cold abscess seen on MRI aid in the diagnosis of osteoarticular tuberculosis. The positive predictive value of MRI for EPTB is close to 96% [24,25]. GeneXpert-MTB (Cepheid, Sunnyvale, California) or cartridge-based nucleic acid amplification test (CBNAAT) for MycoTB™ (Copan Group, Brescia, Italy) is a very sensitive and specific test (93-95%) not only for detection of mycobacterium tuberculosis in samples but also as an aid in detecting anti-microbial resistance. Moreover, the test has a short turnaround time [26,27]. Demonstration of TB bacilli and/ or caseous granulomas from biopsy specimens using ZN staining and fluorescent auramine staining is still a gold standard. However, the yield is low due to the paucibacillary nature of the disease [28]. In geographic zones where TB is highly endemic, a concept of "Trial-ATT" is prevalent. Here, a case with a high probability of TB, based on clinic-radiological assessment but biopsy samples smear-negative, is started on ATT for three weeks, and clinical response noted. However, this is not a norm but an exception, especially when GeneXpert-MTB tests are becoming increasingly part of RNTCP [29]. 

The duration of ATT to be given in OA-TB is also a topic of debate. Current literature suggests ATT be given for 12-18 months with an increased duration especially in children, immune-compromised individuals, and those with concomitant connective tissue disorders [7]. As per RNTCP index guidelines given in 2016, ATT is to be given for a minimum of 12 months with a long-term follow-up of at least 24 months [30].

What is overtly clear is that tuberculosis as a disease entity is continuing unabated in the Asian subcontinent despite tremendous efforts by governments. It is a slowly disappearing disease in developing countries, and a re-emerging entity of the new world, especially due to immunocompromised conditions and diseases [31]. Despite varied occurrence, it continues to intrigue the medical community worldwide. With the emergence of multi-drug resistance (MDR) and extensively-drug resistance (XDR) tuberculosis, the importance of identifying these subtypes is also imperative and important [32]. The GeneXpert-MTB clears the dilemma in the choice of anti-tubercular chemotherapy vis-à-vis drug-resistant tuberculosis.

## Conclusions

Diagnosis of certain cases of osteoarticular tuberculosis may not be simple because of similarity in the clinical or radiological picture with other entities. Pediatric osteoarticular tuberculosis is further intriguing because of its tendency to present at rare sites, multiple foci, or at times because of the spread of infection to adjacent areas. Deeper clinical insight, non-invasive advanced radiological investigations coupled with incisional biopsy and molecular diagnostic tools come to the rescue. However, a high degree of suspicion is a must in all such cases.
